# dl-Asparaginium perchlorate

**DOI:** 10.1107/S1600536809033534

**Published:** 2009-08-29

**Authors:** Fatiha Guenifa, Lamia Bendjeddou, Aouatef Cherouana, Slimane Dahaoui, Claude Lecomte

**Affiliations:** a Laboratoire de Chimie Moléculaire, du Contrôle, de l’Environnement et des Mesures Physico-Chimiques, Faculté des Sciences Exactes, Département de Chimie, Université Mentouri de Constantine, 25000 Constantine, Algeria; b Laboratoire de Cristallographie et Modélisation, des Matériaux Minéraux et Biologiques, (LCM3B), Université Henri Poincaré Nancy I, UPRESA CNRS 7036, BP 239, 54506, Vandoeuvre les Nancy, France

## Abstract

Two enantiomeric counterparts (l- and d-asparginium cations related by glide planes) are present in the structure of the title compound, C_4_H_9_N_2_O_3_^+^·ClO_4_^−^, with a 1:1 cation–anion ratio. The structure is built up from asparginium cations and perchlorate anions. In the crystal, mol­ecules assemble in double layers parallel to (100) through N—H⋯O, O—H⋯O and C—H⋯O hydrogen bonds. In the asparginium layers, hydrogen bonds generate alternating *R*_2_^2^(8) and *R*_4_^3^(18) graph-set motifs. Further hydrogen bonds involving the anions and cations result in the formation of a three-dimensional network.

## Related literature

For the use of dl-asparagine in growth-media for bacteria, see: Gerhardt & Wilson (1948 ▶); Palleroni *et al.* (1973 ▶); van Wagtendonk *et al.* (1963 ▶). For related structures, see: Aarthy *et al.* (2005 ▶); Anitha *et al.* (2005 ▶); Bendjeddou *et al.* (2009 ▶); Verbist *et al.* (1972 ▶); Wang *et al.* (1985 ▶); Yamada *et al.* (2007 ▶). For hydrogen-bond motifs, see: Bernstein *et al.* (1995 ▶).
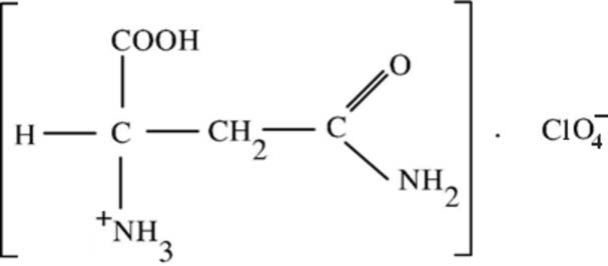


## Experimental

### 

#### Crystal data

C_4_H_9_N_2_O_3_^+^·ClO_4_^−^
*M* *_r_* = 232.58
Orthorhombic, 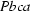

*a* = 9.861 (5) Å
*b* = 10.289 (4) Å
*c* = 16.700 (5) Å
*V* = 1694.4 (12) Å^3^
*Z* = 8
Mo *K*α radiation
μ = 0.47 mm^−1^
*T* = 100 K
0.09 × 0.04 × 0.02 mm

#### Data collection

Oxford Diffraction Xcalibur Saphire2 CCD diffractometer
Absorption correction: none
45509 measured reflections
2818 independent reflections
2205 reflections with *I* > 2σ(*I*)
*R*_int_ = 0.033

#### Refinement

*R*[*F* ^2^ > 2σ(*F* ^2^)] = 0.034
*wR*(*F* ^2^) = 0.100
*S* = 1.12
2818 reflections
127 parameters
1 restraint
H-atom parameters constrained
Δρ_max_ = 0.68 e Å^−3^
Δρ_min_ = −0.38 e Å^−3^

### 

Data collection: *CrysAlis CCD* (Oxford Diffraction, 2008 ▶); cell refinement: *CrysAlis RED* (Oxford Diffraction, 2008 ▶); data reduction: *CrysAlis RED*; program(s) used to solve structure: *SIR92* (Altomare *et al.*, 1993 ▶); program(s) used to refine structure: *SHELXL97* (Sheldrick, 2008 ▶); molecular graphics: *ORTEP-3* (Farrugia, 1997 ▶); software used to prepare material for publication: *WinGX* (Farrugia, 1999 ▶), *PARST97* (Nardelli, 1995 ▶) and *Mercury* (Macrae *et al.*, 2006 ▶).

## Supplementary Material

Crystal structure: contains datablocks global, I. DOI: 10.1107/S1600536809033534/at2865sup1.cif

Structure factors: contains datablocks I. DOI: 10.1107/S1600536809033534/at2865Isup2.hkl

Additional supplementary materials:  crystallographic information; 3D view; checkCIF report

## Figures and Tables

**Table 1:** Hydrogen-bond geometry (Å, °)

*D*—H⋯*A*	*D*—H	H⋯*A*	*D*⋯*A*	*D*—H⋯*A*
O1—H1⋯O3^i^	0.82	1.76	2.5485 (19)	161
N1—H1*A*⋯O4^ii^	0.89	2.02	2.837 (2)	152
N1—H1*B*⋯O5^iii^	0.89	2.03	2.910 (2)	171
N1—H1*C*⋯O3	0.89	2.30	2.886 (2)	123
N1—H1*C*⋯O5	0.89	2.16	2.907 (2)	142
N2—H4*N*⋯O2^iv^	0.84	2.54	3.341 (2)	159
N2—H5*N*⋯O2^v^	0.84	2.57	3.362 (2)	157
N2—H5*N*⋯O5^v^	0.84	2.55	3.089 (2)	123
C2—H2⋯O7^vi^	0.98	2.44	3.201 (2)	134
C3—H3*A*⋯O4^ii^	0.97	2.58	3.326 (2)	134
C3—H3*B*⋯O2^v^	0.97	2.41	3.253 (2)	145

Symmetry codes: (i) 
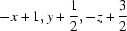
; (ii) 
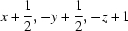
; (iii) 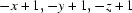; (iv) 
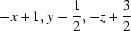
; (v) 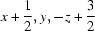; (vi) 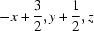.

**Fig. 1.**
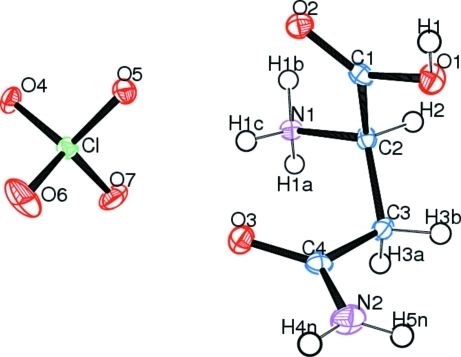
The asymmetric unit of of DL-asparaginium perchlorate, showing the crystallographic numbering scheme. Displacement ellipsoids are drawn at the 50% probability level and H atoms are shown as spheres of arbitrary radii.

**Fig. 2.**
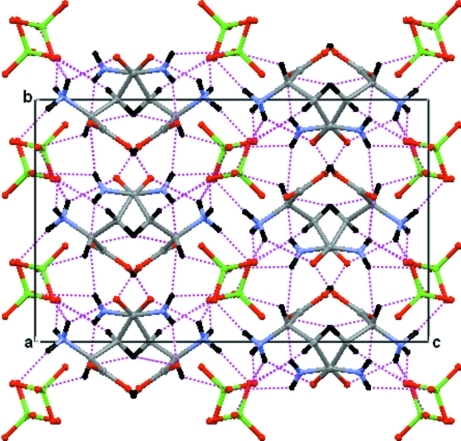
A packing diagram for the title compound, viewed down the a axis, showing the double layers. Dashed lines indicate N—H···O, O—H···O and C—H···O hydrogen bonds

**Fig. 3.**
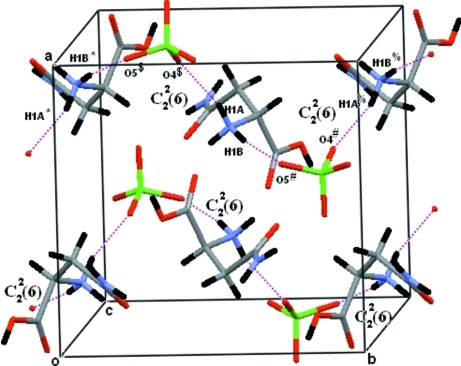
Part of the crystal structure, showing the aggregation of C22(6) motif via N—H···O hydrogen bonds. Atoms marked with a hash symbol (#), dollar sign ($), a percent sign (%), or a star (*) are at the symmetry positions (1 - x, 1 - y, 1 - z), (1/2 + x,1/2 - y, 1 - z), (1.5 - x, 1/2 + y, z), (1.5 - x, -1/2 + y, z) respectively.

**Fig. 4.**
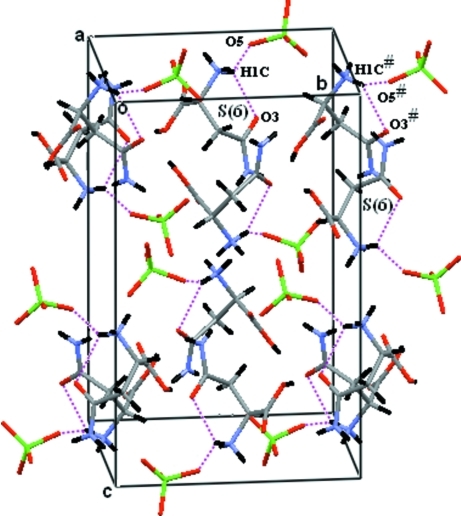
Part of the crystal structure, showing the formation of a finite chaine D(4) and S(6) rings. Atoms marked with an hash symbol (#), are at the symmetry position (x, 1.5 - y,-1/2 + z).

**Fig. 5.**
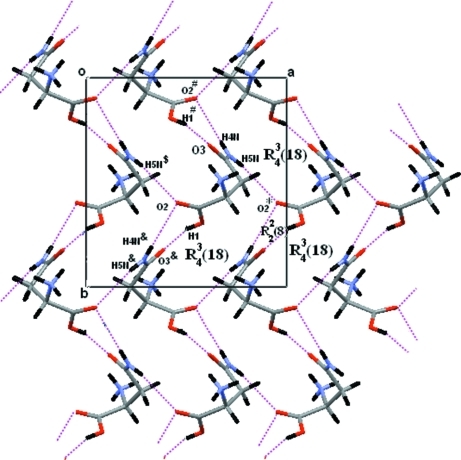
Part of the crystal structure, showing the formation of R22(8) and R43(18) rings. Atoms marked with an ampersand (&), a hash symbol (#), dollar sign ($), or a star (*) are at the symmetry positions (1 - x, 1/2 + y, 1.5 - z), (1 - x, -1/2 + y, 1.5 - z), (-1/2 + x, y, 1.5 - z), (1/2 + x, y, 1.5 - z), respectively.

**Fig. 6.**
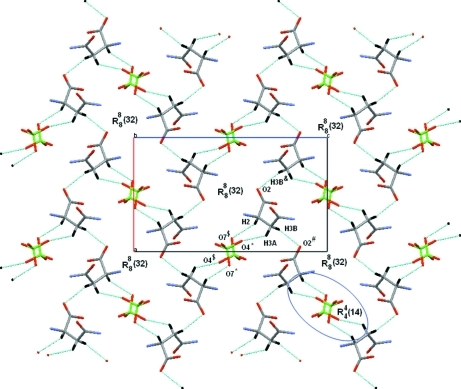
Part of the crystal structure, showing the formation of R88(32) rings. Atoms marked with a star (*), dollar sign ($), an ampersand (&), a hash symbol (#) are at the symmetry positions (1/2 + x, 1/2 - y, 1 - z), (1.5 - x, 1/2 + y, z), (-1/2 + x, y, 1.5 - z), (1/2 + x, y, 1.5 - z) respectively.

## supplementary crystallographic information

### Comment

The asparagine is one of twenty natural amino acids the most common land. DL-asparagine has been used in growth-media for bacteria-growth such as Brucellae (Gerhardt & Wilson, 1948), Pseudomonas fluorescens (Palleroni *et al.*, 1973) and lambda particles (Wagtendonk *et al.*, 1963).

The crystal structure of *L*-asparagine (Yamada *et al.*, 2007), *L*-asparagine monohydrate, (Verbist *et al.*, 1972), *L*-asparagine-*L*-aspartic acid monohydrate (Wang *et al.*, 1985), *L*-asparaginium nitrate (Aarthy *et al.*, 2005) and *L*-asparaginium picrate (Anitha *et al.*, 2005) have been solved. In this paper, the crystal structure information of DL-asparaginium perchlorate at 100 K was undertaken.

The asymmetric unit of (I) (Fig. 1) is formed by a monoprotonated asparaginium cation and a perchlorate anion. A proton transfer from the perchloric acid to atom N(1) of aspargine resulted in the formation of salts. This protonation lead to the different C—O bond distances [1.2179 (18)Å and 1.3086 (18) Å] and bond angle [126.42 (13)°] of the carboxyl group. This type of protonation is observed in various aspargine acid complexes (Anitha *et al.*, 2005; Aarthy, *et al.*, 2005).

The average Cl—O bond distances and O—Cl—O bond angles of the perchlorate anion are 1.4434%A and 109.47 °, respectively, confirming a tetrahedral configuration, similar to other perchlorate studied at low temperature (Bendjeddou *et al.*, 2009).

In (I), the ions are connected *via* N—H···O, O—H···O and C—H···O hydrogen bonds (Table 1) into three-dimensional hydrogen bonded double layers which run parallel to the (100) plane (Fig. 2). All ammonium H atoms are involved in hydrogen bonds, with three different perchlorate ions, while two anions accepts one hydrogen bond. These Two interactions link the anions and cations in to zigzag infinite chains along the [010] direction, which can be described by the graph-set motif C_2_^2^(6) (Bernstein *et al.*, 1995) (Fig. 3). The third anion participate in two centred hydrogen bonds with O(5) atom to form a finite chaine D(4). An intramolecular hydrogen bond is also observed between the α -amino group and the γ-carbonyl group with the graph-set motif S(6) (Fig. 4).

The carboxylic acid H and carbonyl O atoms participates respectively with a neighbouring cation through an O—H···O and N—H···O hydrogen bond. The combination of these hydrogen bonds generates an alternating noncentrosymmetric rings in two-dimensional network which can be described by the graph-set motif *R*_2_^2^(8) and *R*_4_^3^(18) (Fig. 5).

The junction between the cationic entities is consolidated by three weaks independent C—H···O hydrogen bonds *via* the perchlorate anions, forming an *R*^8^_8_(32) and *R*^4^_4_(14) centrosymmetric Rings in two-dimensional network (Fig. 6).

### Experimental

The monocrystals of the compound DL-asparaginium perchlorate are obtained by slow evaporation at room temperature of an aqueous solution containing DL-asparagine monohydrate and the perchloric acid in a 1:1 stochiometric ratio. The solution was maintained in 293 K under agitation during twenty minutes.

### Refinement

H atoms were positioned geometrically and refined in the riding-model approximation, with C—H = 0.98 Å (methine) or 0.97 Å (methylene), N—H = 0.89 Å (ammonium) or 0.84 Å (amine), O—H = 0.82 Å, and with *U*_iso_(H) = 1.2U_eq_(C, N) or 1.5U_eq_(O).

### Crystal data

**Table:**

C_4_H_9_N_2_O_3_^+^·ClO_4_^−^	*F*(000) = 960
*M**_r_* = 232.58	*D*_x_ = 1.823 Mg m^−^^3^
Orthorhombic, *P**b**c**a*	Mo *K*α radiation, λ = 0.71073 Å
Hall symbol: -P 2ac 2ab	Cell parameters from 2818 reflections
*a* = 9.861 (5) Å	θ = 3.1–31.5°
*b* = 10.289 (4) Å	µ = 0.47 mm^−^^1^
*c* = 16.700 (5) Å	*T* = 100 K
*V* = 1694.4 (12) Å^3^	Needle, colourless
*Z* = 8	0.09 × 0.04 × 0.02 mm

### Data collection

**Table:**

Oxford Diffraction Xcalibur Saphire2 CCD diffractometer	2205 reflections with *I* > 2σ(*I*)
Radiation source: fine-focus sealed tube	*R*_int_ = 0.033
graphite	θ_max_ = 31.5°, θ_min_ = 3.1°
φ and ω scans	*h* = −14→14
45509 measured reflections	*k* = −15→11
2818 independent reflections	*l* = −24→24

### Refinement

**Table:**

Refinement on *F*^2^	1 restraint
Least-squares matrix: full	H-atom parameters constrained
*R*[*F*^2^ > 2σ(*F*^2^)] = 0.034	*w* = 1/[σ^2^(*F*_o_^2^) + (0.0538*P*)^2^ + 0.6998*P*] where *P* = (*F*_o_^2^ + 2*F*_c_^2^)/3
*wR*(*F*^2^) = 0.100	(Δ/σ)_max_ = 0.001
*S* = 1.12	Δρ_max_ = 0.68 e Å^−^^3^
2818 reflections	Δρ_min_ = −0.38 e Å^−^^3^
127 parameters	

### Special details

**Table:**

Geometry. Bond distances, angles *etc*. have been calculated using the rounded fractional coordinates. All su's are estimated from the variances of the (full) variance-covariance matrix. The cell e.s.d.'s are taken into account in the estimation of distances, angles and torsion angles
Refinement. Refinement of *F*^2^ against ALL reflections. The weighted *R*-factor *wR* and goodness of fit *S* are based on *F*^2^, conventional *R*-factors *R* are based on *F*, with *F* set to zero for negative *F*^2^. The threshold expression of *F*^2^ > σ(*F*^2^) is used only for calculating *R*-factors(gt) *etc*. and is not relevant to the choice of reflections for refinement. *R*-factors based on *F*^2^ are statistically about twice as large as those based on *F*, and *R*- factors based on ALL data will be even larger.

### Fractional atomic coordinates and isotropic or equivalent isotropic displacement parameters (Å^2^)

**Table:**

	*x*	*y*	*z*	*U*_iso_*/*U*_eq_	
O1	0.58079 (10)	0.69351 (10)	0.73615 (6)	0.0162 (3)	
O2	0.45528 (9)	0.59815 (10)	0.63971 (6)	0.0139 (3)	
O3	0.61640 (11)	0.32442 (10)	0.70705 (6)	0.0158 (3)	
N1	0.67333 (11)	0.48834 (11)	0.57093 (6)	0.0115 (3)	
N2	0.71473 (14)	0.37342 (13)	0.82581 (7)	0.0204 (4)	
C1	0.56293 (14)	0.62144 (13)	0.67245 (8)	0.0117 (3)	
C2	0.69857 (13)	0.56919 (13)	0.64328 (8)	0.0111 (3)	
C3	0.77716 (13)	0.49753 (13)	0.70849 (8)	0.0126 (3)	
C4	0.69625 (14)	0.39103 (13)	0.74834 (8)	0.0127 (3)	
Cl	0.47309 (3)	0.19831 (3)	0.51836 (2)	0.0127 (1)	
O4	0.40552 (10)	0.17287 (10)	0.44286 (6)	0.0158 (3)	
O5	0.43440 (10)	0.32809 (10)	0.54535 (6)	0.0145 (3)	
O6	0.43348 (15)	0.10450 (12)	0.57652 (7)	0.0315 (4)	
O7	0.61761 (11)	0.19650 (11)	0.50614 (7)	0.0226 (3)	
H1	0.50733	0.72120	0.75158	0.0243*	
H1A	0.75164	0.45681	0.55290	0.0172*	
H1B	0.63499	0.53694	0.53310	0.0172*	
H1C	0.61828	0.42291	0.58350	0.0172*	
H2	0.75352	0.64382	0.62663	0.0133*	
H3A	0.85826	0.46000	0.68513	0.0151*	
H3B	0.80548	0.55964	0.74887	0.0151*	
H4N	0.67437	0.31063	0.84737	0.0246*	
H5N	0.77523	0.41807	0.84813	0.0246*	

### Atomic displacement parameters (Å^2^)

**Table:**

	*U*^11^	*U*^22^	*U*^33^	*U*^12^	*U*^13^	*U*^23^
O1	0.0144 (5)	0.0187 (5)	0.0156 (5)	0.0014 (4)	0.0022 (4)	−0.0059 (4)
O2	0.0116 (4)	0.0155 (5)	0.0146 (5)	0.0008 (3)	0.0014 (3)	0.0001 (4)
O3	0.0162 (5)	0.0177 (5)	0.0135 (4)	−0.0048 (4)	−0.0012 (4)	0.0035 (4)
N1	0.0113 (5)	0.0127 (5)	0.0104 (5)	0.0015 (4)	0.0010 (4)	0.0002 (4)
N2	0.0253 (7)	0.0250 (7)	0.0110 (5)	0.0020 (5)	−0.0013 (4)	0.0023 (5)
C1	0.0134 (6)	0.0092 (5)	0.0125 (6)	−0.0007 (4)	0.0028 (4)	0.0024 (5)
C2	0.0104 (5)	0.0108 (5)	0.0120 (5)	−0.0010 (4)	0.0013 (4)	−0.0014 (4)
C3	0.0102 (5)	0.0141 (6)	0.0135 (6)	−0.0005 (4)	−0.0017 (4)	−0.0010 (5)
C4	0.0128 (5)	0.0133 (6)	0.0119 (6)	0.0045 (5)	0.0010 (4)	−0.0002 (5)
Cl	0.0164 (2)	0.0106 (2)	0.0112 (2)	−0.0003 (1)	−0.0021 (1)	0.0004 (1)
O4	0.0155 (5)	0.0178 (5)	0.0142 (5)	−0.0034 (4)	−0.0043 (4)	−0.0027 (4)
O5	0.0165 (5)	0.0125 (5)	0.0145 (5)	0.0006 (3)	−0.0008 (4)	−0.0023 (4)
O6	0.0588 (9)	0.0177 (5)	0.0179 (6)	−0.0056 (5)	0.0043 (5)	0.0082 (4)
O7	0.0145 (5)	0.0267 (6)	0.0265 (6)	0.0061 (4)	−0.0080 (4)	−0.0079 (4)

### Geometric parameters (Å, °)

**Table:**

Cl—O5	1.4601 (13)	N1—H1C	0.8900
Cl—O6	1.4239 (15)	N1—H1B	0.8900
Cl—O4	1.4499 (13)	N2—H4N	0.8400
Cl—O7	1.4398 (13)	N2—H5N	0.8400
O1—C1	1.3086 (18)	C1—C2	1.522 (2)
O2—C1	1.2179 (18)	C2—C3	1.527 (2)
O3—C4	1.2511 (18)	C3—C4	1.510 (2)
O1—H1	0.8200	C2—H2	0.9800
N1—C2	1.4879 (19)	C3—H3A	0.9700
N2—C4	1.3190 (19)	C3—H3B	0.9700
N1—H1A	0.8900		
			
O5—Cl—O6	109.74 (7)	O1—C1—C2	110.01 (11)
O5—Cl—O7	108.32 (6)	O1—C1—O2	126.42 (13)
O6—Cl—O7	111.07 (8)	N1—C2—C3	113.22 (11)
O4—Cl—O5	108.27 (6)	N1—C2—C1	108.09 (10)
O4—Cl—O6	110.17 (7)	C1—C2—C3	112.86 (11)
O4—Cl—O7	109.23 (6)	C2—C3—C4	113.37 (11)
C1—O1—H1	109.00	N2—C4—C3	117.32 (12)
C2—N1—H1A	109.00	O3—C4—N2	123.53 (13)
C2—N1—H1B	109.00	O3—C4—C3	119.16 (12)
H1B—N1—H1C	109.00	N1—C2—H2	107.00
H1A—N1—H1C	109.00	C1—C2—H2	107.00
C2—N1—H1C	109.00	C3—C2—H2	107.00
H1A—N1—H1B	109.00	H3A—C3—H3B	108.00
C4—N2—H5N	117.00	C2—C3—H3A	109.00
H4N—N2—H5N	125.00	C2—C3—H3B	109.00
C4—N2—H4N	117.00	C4—C3—H3A	109.00
O2—C1—C2	123.57 (12)	C4—C3—H3B	109.00

### Hydrogen-bond geometry (Å, °)

**Table:**

*D*—H···*A*	*D*—H	H···*A*	*D*···*A*	*D*—H···*A*
O1—H1···O3^i^	0.8200	1.7600	2.5485 (19)	161.00
N1—H1A···O4^ii^	0.8900	2.0200	2.837 (2)	152.00
N1—H1B···O5^iii^	0.8900	2.0300	2.910 (2)	171.00
N1—H1C···O3	0.8900	2.3000	2.886 (2)	123.00
N1—H1C···O5	0.8900	2.1600	2.907 (2)	142.00
N2—H4N···O2^iv^	0.8400	2.5400	3.341 (2)	159.00
N2—H5N···O2^v^	0.8400	2.5700	3.362 (2)	157.00
N2—H5N···O5^v^	0.8400	2.5500	3.089 (2)	123.00
C2—H2···O7^vi^	0.9800	2.4400	3.201 (2)	134.00
C3—H3A···O4^ii^	0.9700	2.5800	3.326 (2)	134.00
C3—H3B···O2^v^	0.9700	2.4100	3.253 (2)	145.00

Symmetry codes: (i) −*x*+1, *y*+1/2, −*z*+3/2; (ii) *x*+1/2, −*y*+1/2, −*z*+1; (iii) −*x*+1, −*y*+1, −*z*+1; (iv) −*x*+1, *y*−1/2, −*z*+3/2; (v) *x*+1/2, *y*, −*z*+3/2; (vi) −*x*+3/2, *y*+1/2, *z*.
